# Transcriptome Sequencing and Identification of *APOE* Gene Polymorphisms, Their Expression and Their Relationship with Body Size Traits in Guizhou White Goats (*Capra hircus*)

**DOI:** 10.3390/ani16071031

**Published:** 2026-03-27

**Authors:** Wen-Ying Wang, Lin-Guang Dai, Jun-You Huang, Xing-Chao Song, Jin-Zhu Meng, Yuan-Yuan Zhao, Zhen-Yang Wu, Qing-Ming An

**Affiliations:** Guizhou Provincial Key Laboratory for Biodiversity Conservation and Utilization in the Fanjing Mountain Region, College of Agriculture and Forestry Engineering, Tongren University, Tongren 554300, China; w19985578865@163.com (W.-Y.W.); 17385660148@163.com (L.-G.D.); 16685142101@163.com (J.-Y.H.); songxingchao_888@126.com (X.-C.S.); mjz122021@126.com (J.-Z.M.); 84840293@163.com (Y.-Y.Z.); wuzhenyang0724@163.com (Z.-Y.W.)

**Keywords:** *APOE* gene, nucleotide variation, body size traits, sex-specific, goat

## Abstract

Carcass growth and development are crucial evaluation indicators influencing the economic efficiency of goats (*Capra hircus*). This study aimed to screen the nucleotide variation sites (SNPs) of the *APOE* gene in Guizhou white goats and explore the correlation between *APOE* gene variations and body size traits, as *APOE* had been identified as a key candidate gene regulating growth and development in this breed through transcriptome sequencing screening. A total of 324 Guizhou white goats were used in this study for SNP detection, population genetic analysis, real-time fluorescence quantitative PCR (RT-qPCR) and association analysis. The results showed that one nucleotide mutation site (g.353 A > G) was detected in the *APOE* gene, which yielded two alleles (A and G) and three genotypes (AA, AG and GG). The site exhibited moderate polymorphism and conformed to Hardy–Weinberg equilibrium. The mRNA expression level of *APOE* in longissimus dorsi muscle was significantly higher in males than in females. Association analysis revealed a sex-specific effect of this locus on body size traits. The A allele and AA genotype were significantly associated with increased body weight and heart girth in females, whereas no significant effect was detected in males. Therefore, the identified *APOE* gene mutation site can serve as a candidate molecular marker for the early selection of growth traits in Guizhou white goats.

## Simple Summary

Apolipoprotein E (APOE) is a key regulatory protein involved in lipid metabolism, and previous studies have demonstrated its significant association with meat quality and meat yield in livestock. In this study, a specific variation in the *APOE* gene was found to significantly improve body size traits in female goats, whereas no significant effects were observed in males. These findings suggest that this genetic locus could serve as a candidate molecular marker for the selective breeding of female goats with superior body size traits. Overall, our results indicate that the *APOE* gene represents a promising candidate molecular marker for the marker-assisted selection of growth traits in Guizhou white goats, providing a genetic basis for improving their production performance.

## 1. Introduction

Apolipoproteins are protein constituents of plasma lipoproteins and play essential roles in lipid transport and metabolism. To date, more than 20 types of apolipoproteins have been identified, which are primarily classified into families including APOA, APOB, APOD, APOE, APOH and APOM based on their structural and functional properties. Among these, apolipoprotein E (APOE) is a key regulatory protein involved in lipid metabolism in animals. Synthesized in the liver, the spleen, the kidney, and other tissues, APOE enters the blood circulation, distributes widely throughout the body, participates in the transport of cholesterol, phospholipids, and triglycerides, and plays a critical role in lipid metabolism [1,2]. In humans, the *APOE* gene is mapped to the q13.2 region on chromosome 19. It consists of four exons and three introns, encoding a total of 299 amino acids [3,4,5]. In goats, the *APOE* gene is mapped on chromosome 18 and consists of four exons and three introns, encoding a total of 316 amino acids. The APOE protein plays a central role in maintaining lipid homeostasis in both the central nervous system (CNS) and peripheral tissues, mediating the intracellular uptake of lipoproteins primarily through binding to the low-density lipoprotein receptor (LDLR) family [1,4,6]. More importantly, the *APOE* gene displays abundant allelic polymorphism. Notably, the three major alleles (ε2, ε3, and ε4), which arise from specific single-nucleotide polymorphisms (SNPs), exhibit distinct biological functions [5,7,8]. Some studies have demonstrated that different *APOE* isoforms not only are associated with various diseases, but also exhibit antioxidant, anti-inflammatory and anti-atherosclerotic effects [7]. Polymorphisms of the *APOE* gene contribute to susceptibility to multiple conditions, including epilepsy, non-alcoholic fatty liver disease (NAFLD) and cardiovascular diseases [1,5,9,10,11,12]. Using somatic cell hybridization and fluorescence in situ hybridization (FISH) techniques, researchers have localized the human *APOE* gene to a chromosomal region that also contains other lipid metabolism-related genes, such as apolipoprotein CI (APOCI) [7]. Sukalskaia demonstrated that APOE can specifically interact with tweety homolog protein 2 (TTYH2) in endosomes, thereby co-mediating lipid transport [13]. These findings indicate that the *APOE* gene can synergistically regulate lipid homeostasis through its interacting genes.

In peripheral tissues, studies have further demonstrated that APOE is predominantly synthesized in the liver, adrenal glands and adipose tissues, where it participates in systemic lipid metabolism. In the liver, APOE helps maintain plasma cholesterol and triglyceride levels by regulating the metabolism of very-low-density lipoprotein (VLDL) and high-density lipoprotein (HDL) [14]. Specifically, APOE3 promotes the conversion of VLDL to low-density lipoprotein (LDL), whereas APOE4 exhibits excessively strong binding affinity for the LDL receptor (LDLR), which impairs hepatic LDL uptake and increases the risk of atherosclerosis [1,15]. In adipose tissues, APOE influences obesity susceptibility by modulating adipocyte differentiation and lipid storage. Specifically, individuals carrying the APOE4 allele exhibit accelerated adipocyte lipolysis, which may contribute to a low-fat body weight phenotype [14,16]. In contrast, APOE2 is associated with increased lipid accumulation in adipocytes, thereby predisposing carriers to obesity-related metabolic disorders [17,18].

In animal production and livestock breeding, polymorphisms in the *APOE* gene have been significantly associated with meat quality traits, such as marbling in beef and intramuscular fat content in pork. This suggests that the *APOE* gene is a promising candidate gene for marker-assisted selection (MAS). Daniels et al. [14] reported a significant association between the *APOE* gene g.11400G > A polymorphism and subcutaneous fat depth (SFD) in Wagyu×Limousin crossbred cattle (*p* < 0.05). Zhao et al. [19] demonstrated that *APOE* gene expression in the adipose tissue of Congjiang Xiang pigs was second only to that in the liver, and was positively correlated with intramuscular fat content (r = 0.42, *p* < 0.05). These findings suggest that the *APOE* gene could serve as a candidate molecular marker for fat deposition traits in livestock.

Despite extensive research on the *APOE* gene, most studies have primarily focused on its association with diseases. However, the regulatory mechanisms by which *APOE* gene variation influences obesity in mammals remain largely unclear. These include, for example, the tissue-specific functional roles of the *APOE* gene, such as the differences between central astrocytes and peripheral hepatocytes, the molecular pathways through which allelic polymorphisms influence diseases, such as how ε4 promotes neuroinflammation via lipid metabolism disorders, and the modifying effects of sex and age on APOE function [10]. In summary, the *APOE* gene plays a crucial role in regulating animal growth. However, relatively few studies on the *APOE* gene have been conducted in goats to date. Based on transcriptome sequencing analysis, this study aimed to investigate whether the *APOE* gene expression in the longissimus dorsi muscle of goats exhibits sex specificity, and to explore the effects of *APOE* gene variation on goat body size traits. The ultimate goal is to identify molecular genetic markers for the early selection of goats with superior growth traits.

## 2. Materials and Methods

### 2.1. Animals and Data Collection

To investigate genetic variation in the goat *APOE* gene, a total of 324 blood samples were collected and transported to the laboratory, where they were stored at −20 °C. Of these, 100 blood samples were obtained from 7 different farms for nucleotide mutation detection. The DNA from these samples was diluted to a uniform concentration using TE buffer, and 2 µL of each was pooled to create a mixed DNA sample for subsequent pooled sequencing. The remaining 224 samples were collected from 2 cooperative farms of the same company in Tongren City, Guizhou Province, China. On each farm, goats were randomly selected, and all were approximately 2 to 2.5 years of age. Basic body size trait data, including body weight, heart girth, wither height, body length, and circumference of the cannon bone, were recorded for each corresponding individual and used for subsequent association analyses. All genomic DNA was extracted using a blood DNA extraction kit (Tiangen Biotech, Beijing, China). The OD_260_/OD_280_ ratios of the DNA ranged from 1.6 to 1.8, indicating acceptable quality for downstream applications. After passing quality inspection, all DNA samples were uniformly diluted to a concentration of 10 ng/μL. The extracted DNA could either be immediately used for subsequent experiments or be stored at −20 °C.

Additionally, six healthy 2-year-old Guizhou white goats (3 males and 3 females) were selected from the same population under identical feeding conditions, and they were not part of the 224 goats used for association analysis. After the experiment was reviewed and approved by the Ethics Committee of Tongren University, 6 experimental goats were slaughtered in strict accordance with the national standards for experimental animal slaughter, and slaughter performance traits were quantified, including pre-slaughter live weight, carcass weight, meat weight, calculated yield of carcass and meat rate. The detailed slaughter performance data are presented in Table 1. During the slaughter process, longissimus dorsi muscle tissue samples were collected from each of the 6 goats, immediately snap-frozen in liquid nitrogen, transported to the laboratory, and stored at −80 °C for subsequent transcriptome sequencing screening and RT-qPCR assays. Total RNA was isolated from longissimus dorsi tissue using the RNA simple Total RNA Kit (TIANGEN Biotech, Beijing, China) according to the manufacturer’s instructions. Aliquots of 200 ng of total RNA were used for cDNA synthesis with the TransScript One Step gDNA removal and cDNA Synthesis SuperMix (TransGen Biotech, Beijing, China), following the manufacturer’s protocols.

### 2.2. Transcriptome Sequencing and Analysis

Library preparation and subsequent transcriptome sequencing were conducted on the Illumina HiSeq 2500 platform by Beijing Novogene Bioinformatics Technology Co., Ltd, Beijing, China. The quality of raw sequencing data was assessed using the Fastp software (version 0.23.4). Clean data (clean reads) were obtained by removing reads containing adapter. Reference genome and gene model annotation files were downloaded from the genome website. HISAT2 (version 2.2.1) was used to obtain the mapping positions of the assembled sequencing reads on the reference genome as well as the sequence characteristics of the samples (Table 2).

**Figure 1:**
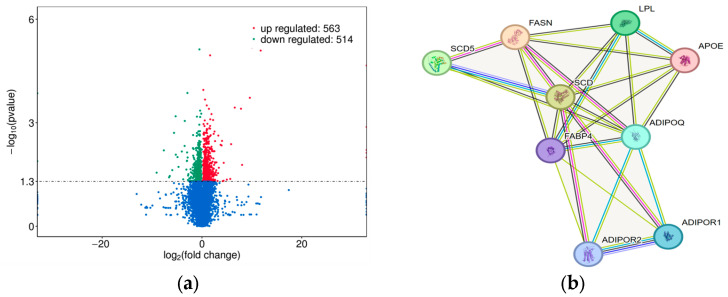
(**a**) Volcano plot of differentially expressed genes in male and female goat longissimus dorsi muscle; (**b**) PPI network of lipid growth-related genes in livestock.

Transcript assembly, annotation, and merging were performed using Cufflinks software (version 2.2.1). FPKM values were used as the metric for quantifying transcript and gene expression levels, and a total of 25,089 expressed genes were identified across the six samples. Analysis of these 25,089 mRNAs was conducted using the DESeq2 R package (version 1.42.0) with the following thresholds: FPKM ≥ 1, MF-FPKM/FL-FPKM > 1, and *p* adjust < 0.05. This analysis yielded a total of 1077 differentially expressed genes (DEGs), of which 563 genes were significantly upregulated and 514 genes were significantly downregulated in the longissimus dorsi muscle of male goats compared to female goats (Figure 1a). Further, through targeted screening of genes previously identified by our research team, as well as those reported in other studies to influence carcass development in ruminants, we found that *APOE*, along with nine other genes, was differentially expressed (Table 3). Subsequently, protein–protein interaction (PPI) network analysis was performed between the *APOE* gene and several lipid growth-related genes previously studied by our research group and others in livestock (Figure 1b), to evaluate the feasibility of conducting an association analysis between the *APOE* gene and body size traits in goats.

### 2.3. Primer Design and PCR Amplification

Using the *APOE* gene sequence information for goats from the NCBI Gene Database (GenBank accession number: NC_030825.1, gene ID: 102170314) as a reference, Primer 5.0 software (Version 5.0, Premier Biosoft International, Palo Alto, CA, USA) was used to design PCR primers targeting partial sequences of intron 1 and exon 3 of the goat *APOE* gene. The expected amplicon sizes were 521 bp and 585 bp. The primers were synthesized by Sangon Biotech (Chengdu, China), dissolved in ultrapure water and stored at −20 °C; primer information is shown in Table 3.

The PCR reaction was performed in a 40 μL volume containing 2 µL of DNA template (1 μg/L), 22 µL of 2 × Taq PCR StarMix, 2 μL each of forward and reverse primers (10 μmol/L) and 12 µL of ddH_2_O. The thermal cycling conditions were as follows: initial denaturation at 95 °C for 5 min, 42 cycles of denaturation at 95 °C for 30 s, annealing at 60 °C for 30 s and extension at 72 °C for 30 s, followed by a final extension at 72 °C for 5 min and holding at 10 °C. Products were analyzed by 1.5% agarose gel (nucleic acid dye added) electrophoresis for 15 min.

RT-qPCR was performed to detect the relative expression level of the *APOE* gene using a fluorescent quantitative PCR detection kit produced by TransGen Biotech Co., Ltd. (Beijing, China). Each 20 µL reaction system contained 1 µL cDNA template (1 µg/L), 10 µL 2 × PCR SuperMix, 0.5 µL each of forward and reverse primers (10 µmol/L), and 8 µL ddH_2_O. The thermal cycling conditions were: 94 °C for 1 min, 38 cycles of 94 °C for 10 s, 59 °C for 30 s and 72 °C for 10 s. Three technical repetitions were performed for each sample, and the results were normalized to the expression level of the glyceraldehyde-3-phosphate dehydrogenase gene (*GAPDH*). Primer information for both PCR and RT-qPCR is provided in Table 4.

### 2.4. Measurements and Statistical Analysis

Mutation sites of the *APOE* gene were identified using MegAlign software within the DNASTAR Lasergene package (Version 7.1.0, DNASTAR, Inc., Madison, WI, USA). Genetic diversity parameters, including genotype frequencies, allele frequencies, and Hardy–Weinberg equilibrium (*χ*^2^ test), were calculated using PopGene 32.0 software (Molecular Biology and Biotechnology Center, University of Alberta, Edmonton, AB, Canada). The polymorphic information content (PIC) was computed using PIC_CALC software (Version 0.6, Yellow Sea Fisheries Research Institute, Chinese Academy of Fishery Sciences, Wuhan, China).

Relative gene expression levels were calculated using the 2^−△△Ct^ method. The ^△△^Ct value was determined as follows: ^△△^Ct = (Ct*^APOE gene^* − Ct*^GAPDH^*) for the experimental group and (Ct*^APOE gene^* − Ct*^GAPDH^*) for the control group. Statistical significance was assessed by one-way ANOVA using SPSS 22.0 software, with *p*-value < 0.05 considered statistically significant.

General linear mixed-effect models (GLMMs) implemented in MINITAB (Version 16, Minitab Inc., State College, PA, USA) were used to evaluate the association between *APOE* alleles and body size traits. For genotypes with a frequency greater than 5%, a series of GLMMs was constructed to assess the effect of each genotype on the body size traits. Multiple pairwise comparisons among *APOE* genotypes were performed using Tukey’s test with Bonferroni correction. In the GLMM analysis of alleles and genotypes in the mixed-sex population, sex was included as a fixed explanatory factor. However, in the sex-specific analyses, sex was not included in the model. Least squares means were compared using the following model:

Y_imn_ = µ + C_i_ + F_m_ + X_n_ + e_imn_

Among them, Y_imn_ represents the phenotypic value of the production trait, µ is the overall population mean, C_i_ is the fixed effect of sex, F_m_ is the fixed effect of genotype or allele, X_n_ represents the interaction effect between different factors, and e_imn_ represents the random error. All results are presented as “mean ± standard error”.

## 3. Results

### 3.1. Analysis of the Polymorphism of the APOE Gene

**Figure 2:**
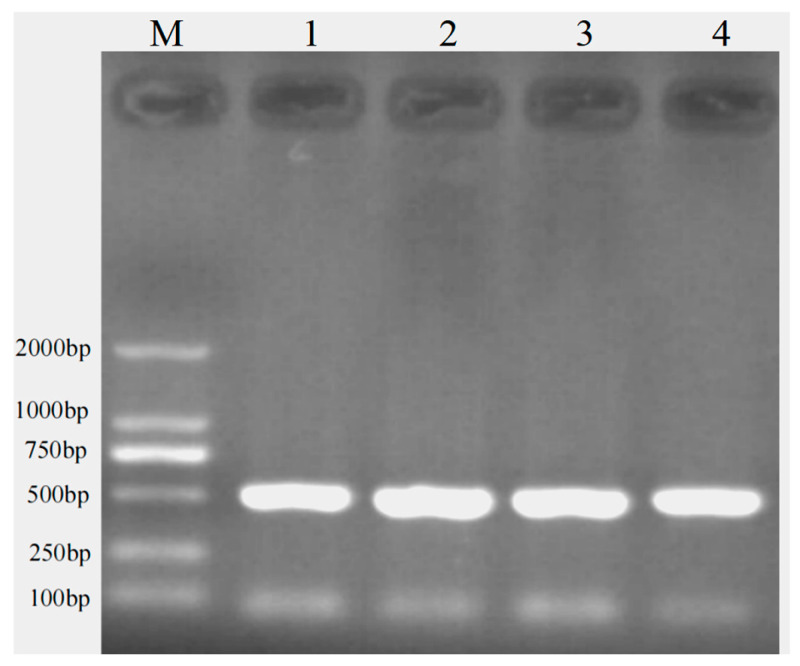
Agarose electrophoresis results of *APOE* intron 1.

**Figure 3:**
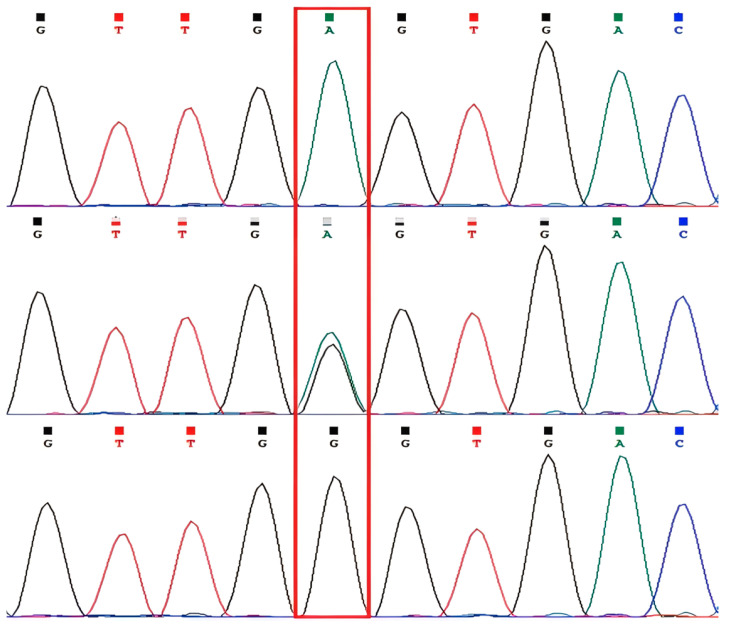
Sequencing chromatograms of the novel SNP in the goat *APOE* gene. The red boxes indicate the nucleotide variation sites.

PCR products were detected by electrophoresis on 1.5% agarose gels. The amplified bands obtained with primer 1 were clear and specific, indicating their suitability for subsequent analyses (Figure 2). Only one nucleotide variation site (g.353 A > G mutation) was identified within the intron 1 fragment (Figure 3). Alignment with the reference sequence localized this variant to intron 1 of the *APOE* gene (corresponding to g.3104 of NC_030825.1; gene ID: 102170314). No variant sites were detected in the exon 2 region amplified by primer 2; therefore, these results are not presented.

The 100 samples from seven different farms were used only for variant detection via pooled sequencing; therefore, population genetic parameters were not analyzed for this sample set. In the 224 Guizhou white goats, three genotypes were identified at the g.353 A > G locus: AA (*n* = 29, 12.97%), AG (*n* = 92, 41.00%) and GG (*n* = 103, 46.03%). The corresponding allele frequencies were 33.47% for A and 66.53% for G. The polymorphic information content (PIC) was calculated as 0.35, falling within the moderate-polymorphism range (high: PIC > 0.5; moderate: 0.25 < PIC < 0.5; low: PIC < 0.25). The chi-square (*χ*^2^) test confirmed that the genotype distribution conformed to Hardy–Weinberg equilibrium (*p* > 0.05). Detailed results are summarized in Table 5.

### 3.2. Differential Expression of APOE Gene in Male and Female Goats

**Figure 4:**
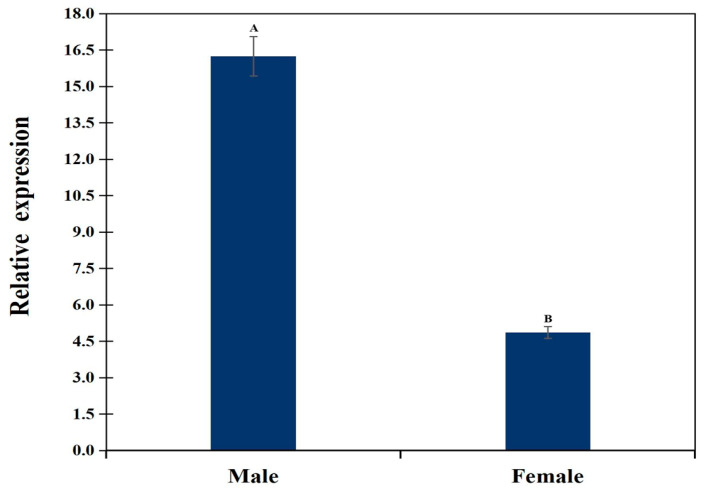
Relative mRNA expression of *APOE* in longissimus dorsi muscle. Different letters above bars indicate significant differences (*p* < 0.05).

The mRNA expression level of the *APOE* gene in longissimus dorsi tissue of male and female goats was detected by RT-qPCR. The results revealed sex-specific expression, with significantly lower expression in females than in male goats (Figure 4).

### 3.3. Effect of Variation in APOE on Body Size Traits

Analysis of the 224 white goats used for association analysis revealed that the frequencies of both alleles (A and G) exceeded 5%, indicating their suitability for association analysis with body size traits. Allele presence/absence analysis revealed that different alleles had no significant effects on heart girth, wither height and circumference of cannon bone, but did significantly affect body weight and body length. Specifically, individuals carrying the A allele exhibited significantly higher body weight and body length compared to non-carriers (*p <* 0.05, Table 6). Genotype association analysis similarly revealed that genotypes significantly influenced body weight and body length, with no significant differences observed for heart girth, wither height and circumference of cannon bone. For body weight and body length, individuals with the AA genotype showed significantly higher values than those with AG and GG genotypes (Table 7).

Allele presence/absence analysis in male and female Guizhou white goats demonstrated a sex-specific effect of the *APOE* gene variant on body size traits. In females, the presence of the A allele was significantly associated with higher body weight and heart girth compared to its absence (*p* < 0.05). In contrast, no significant effects on any traits were observed in male goats (*p* > 0.05, Table 8). The genotype analysis further supported this sex-specific pattern. Female goats carrying the AA genotype exhibited significantly greater body weight and heart girth than those with AG and GG genotypes, while no significant genotype effects were detected in males (Table 9).

## 4. Discussion

The Guizhou white goat is a renowned local breed and an important genetic resource for animal husbandry development in Guizhou Province, China. In the preliminary stage of this experiment, longissimus dorsi muscle samples were collected from male and female goats for transcriptome sequencing analysis. The selected goats were of the same age but differed in body weight, and all were raised under identical feeding conditions. A total of 1077 DEGs associated with carcass muscle growth and development were identified by transcriptome sequencing, with 563 upregulated and 514 downregulated in male relative to female goats. These differentially expressed genes are associated with longissimus dorsi muscle growth and development in Guizhou white goats and warrant further investigation.

Further screening of the 1077 differentially expressed genes, combined with findings from existing studies, led to the selection of 10 candidate genes potentially associated with carcass growth and development in goats. Our research team has conducted further studies on some of these genes and found that the *APOE* gene interacts with those previously investigated. In previous studies, we demonstrated that nucleotide variations in the *MYH2* and *FHL3* genes significantly affect growth performance in Guizhou white goats. Additionally, we found that both the expression and nucleotide variation of the *FASN* gene exert sex-specific effects on body size traits in this breed. Existing research has shown that the APOE protein is a key regulator of lipid metabolism in mammals, widely involved in the transport and metabolism of cholesterol, phospholipids and triglycerides [2,20]. In mice, *APOE* gene knockout leads to progressive lipid metabolism disorders and, consequently, the development of atherosclerotic lesions [21]. Mahley et al. reported that the APOE protein binds to lipids to form soluble discoidal lipoprotein. When triglycerides and cholesterol ester increase, these lipoprotein complexes can be encapsulated by APOE and subsequently taken up by cells via binding to cell membrane receptors [22]. Studies have also shown that the *APOE* gene plays a crucial role in the clearance of low-density lipoprotein (LDL) and chylomicron remnants via the low-density lipoprotein receptor (LDLR) [10]. Given the established roles of APOE protein in regulating lipid metabolism, this study selected the *APOE* gene to examine how its expression and nucleotide variation differentially influence body size traits in Guizhou white goats. This research seeks to provide a scientific basis for enhancing the economic value and sustainability of the Guizhou white goat industry.

Previous studies have demonstrated that the *APOE* gene exhibits significant allelic polymorphism, with three major alleles (ε2, ε3, ε4) that affect lipid metabolism and are associated with various health conditions [5,7,8]. In this study, a nucleotide variation site was identified in intron 1 of the *APOE* gene in Guizhou white goats. Although this mutation does not directly alter the composition or structure of the APOE protein, previous studies have identified enhancer regulatory elements within intron 1 of the *APOE* gene. This nucleotide variation may disrupt the regulatory activity of the enhancer element, potentially leading to altered expression of the *APOE* gene [11]. Furthermore, sequence alignment indicated that intron 1 of the *APOE* gene resides within the promoter region, highlighting its significance for future research. PIC analysis revealed that this nucleotide variation is moderately polymorphic in the Guizhou white goat population (0.25 < PIC < 0.5). Notably, despite a history of long-term selection breeding, the population continues to exhibit substantial genetic diversity and has not deviated from Hardy–Weinberg equilibrium. This indicates that Guizhou white goats retain considerable genetic potential for further improvement in growth rate, meat yield, and meat quality.

The present study revealed sex-specific expression of the *APOE* gene in the longissimus dorsi muscle of Guizhou white goats. This finding is consistent with previous reports in humans, where plasma APOE protein concentrations have been shown to correlate with triglyceride-rich lipoproteins, as well as with age and sex [23]. Within the human reproductive system, the expression of *APOE* in oocytes differs between males and females. Specifically, in female oocytes, APOE protein levels fluctuate with age and are distinct from those associated with plasma lipoprotein complexes [7,24]. Bharati et al. [25] demonstrated that the *APOE* gene plays a critical regulatory role in porcine follicular growth and maturation, with consequent effects on female reproductive performance. In male reproductive cells, the APOE protein is associated with semen lipid content [26,27], potentially through its relationship with total cholesterol levels, which may affect steroid production and consequently influence male reproductive performance [28].

In livestock, studies have also shown that *APOE* gene expression is associated with adipocyte differentiation and fat deposition. Du et al. [29] reported that *APOE* gene expression in subcutaneous fat of 1-year-old goats was the highest among all tissues examined and was significantly higher than that in the heart, the liver, the muscle and other tissues. In pigs, *APOE* gene expression was highest in primary fetal fibroblasts and gradually decreased with increased cell passages, reaching the lowest level in the 50th generation. This suggests that the *APOE* gene exhibits selective expression in fibroblasts [30]. Song Yaping et al. [31] found that the *APOE* gene serves as a key regulatory factor linking muscle development and fat accumulation, and that interference with *APOE* expression can regulate skeletal muscle myogenic differentiation and lipid metabolism. In this study, *APOE* gene expression levels in the longissimus dorsi muscle were significantly higher in male goats than in female goats. Given that APOE protein is a key protein regulator of lipid metabolism in mammals, widely involved in the transport and metabolism of cholesterol, phospholipids and triglycerides [1,2], the elevated expression of the *APOE* gene in males may be associated with accelerated muscle or fat tissue growth. In production practice, male goats typically exhibit higher growth rates and meat yield than females during the rapid growth phase. This observation aligns with our findings of elevated *APOE* expression in males, suggesting a correlation between *APOE* expression and growth traits. Therefore, the *APOE* gene may serve as an important candidate gene regulating growth traits in goats.

Further research revealed that the g.353 bp A > G nucleotide mutation identified in this study significantly affected the body size traits, specifically body weight and body length, in Guizhou white goats. Individuals carrying the A allele exhibited significantly higher body weight and body length compared to non-carriers. Moreover, this effect was sex-specific. Female goats carrying the A allele exhibited higher body weight and heart girth compared to non-carriers, whereas no significant effects were observed for any traits in male goats. Previous studies have also demonstrated significant association between the *APOE* gene and growth traits as well as meat quality traits in animals. Daniels et al. [15] reported that the 11,400 G > A polymorphism in the *APOE* gene was significantly associated with subcutaneous fat depth (SFD) in Wagyu × Limousin crossbred cattle. Zhao et al. [19] reported that the *APOE* gene expression levels in adipose tissue of Congjiang Xiang pigs were positively correlated with intramuscular fat content. Vincent-Viry et al. [25] also found that the concentration of the *APOE* gene was associated with the sex and age of animals. In female oocytes, APOE protein differs from lipoprotein complexes of different densities, whereas in male germ cells, the relationship between APOE protein and total cholesterol levels affects steroid production, thereby influencing reproductive performance [7,24,25,26]. Additionally, the single-nucleotide polymorphism (SNP, rs440446) in the *APOE* gene subtype APOE4 significantly increases the risk of biliary malignant tumors in males, while the SNP (rs1003723) in the APOE receptor *LDLR4* is associated with an elevated risk of cholangiocarcinoma in both sexes, also exhibiting sex-specific effects [32]. In summary, these findings indicate that the *APOE* gene exhibits sex specificity in regulating various physiological functions in animals, which is consistent with the results of the present study.

## 5. Conclusions

In conclusion, this study identified a g.353 A > G mutation in intron 1 of the *APOE* gene in the Guizhou white goat that significantly influences body weight and body length. Both the phenotypic effects of this variant and its expression in the longissimus dorsi muscle were sex-specific. Thus, this SNP can serve as a candidate molecular marker for sex-specific breeding of body size traits in Guizhou white goats and warrants further in-depth investigation.

## Figures and Tables

**Table 1:** Slaughter performance differences between males and females of Guizhou white goats.

Indexes	Ram	Ewe	*p*-Value
Live weight before slaughter (kg)	41.05 ± 3.64	26.12 ± 3.64	0.027
Carcass weight (kg)	19.50 ± 1.86	12.17 ± 1.86	0.032
Yield of carcass (%)	47.35 ± 0.33	46.55 ± 0.32	0.135
Meat weight (kg)	6.81 ± 0.70	4.12 ± 0.70	0.036
Yield of meat (%)	34.78 ± 0.29	33.85 ± 0.29	0.068

**Table 2:** RNA-Seq and mapping to the reference genome.

Sample	Clean Base Data (Gb)	Total Reads	Mapped Reads	Unique Mapped Reads	Multiple Mapped Reads	Q30/%
M1	13.47	83,030,104	78,703,723 (94.79%)	69,863,817 (84.14%)	8,839,906 (10.65%)	89.86
M2	12.90	95,739,024	90,167,003 (94.18%)	82,561,093 (86.24%)	7,605,910 (7.94%)	90.42
M3	13.25	83,704,190	78,475,828 (93.75%)	72,994,411 (87.21%)	5,481,417 (6.55%)	89.97
F1	12.45	89,806,996	84,783,459 (94.41%)	77,295,726 (86.07%)	7,487,733 (8.34%)	90.81
F2	14.36	86,000,412	81,287,190 (94.52%)	74,457,221 (86.58%)	6,829,969 (7.94%)	92.86
F3	12.56	88,311,882	82,976,580 (93.96%)	74,701,217 (84.59%)	8,275,363 (9.37%)	92.77

Note: M represents male; F represents female.

**Table 3:** Ten candidate genes that may be associated with longissimus dorsi tissue development of goats with different sexes.

Gene Name	Male FPKM	Female FPKM	*p*-Value
*FASN* (fatty acid synthase)	2.77	0.17	<0.01
*APOE* (apolipoprotein E)	17.43	5.99	<0.01
*CPXM1* (Carboxy peptidase X, M14 family member 1)	8.41	2.50	<0.01
*HOXD9* (Homeobox D9)	17.91	9.64	<0.01
*MYH2* (Myosin Heavy Chain 2)	372.09	248.39	<0.01
*FHL3* (four and a half LIM domain protein 3)	116.69	24.86	<0.01
*CTTN* (Cortactin)	10.72	13.13	<0.01
*MSTN* (Myostatin)	19.38	3.74	0.02
*PRKAG3* (protein kinase AMP-activated non-catalytic subunit gamma 3)	16.35	7.16	0.03
*WFIKKN2* (WAP, follistatin/kazal, immunoglobulin, kunitz and netrin domain-containing 1)	2.86	0.89	0.04

**Table 4:** Primer information used for PCR and RT-qPCR.

Primers	Primer Sequences (5′→3′)	Tm (°C)	Product Length (bp)
*APOE-1* (Intron 1)	F:5′-GCGGAAGACAGCGTTTAG-3′	60	521
	R:5′-CGGACCACGGACGGGAGGACGACAA-3′		
*APOE-2* (Exon 3)	F:5′-TGGAGCACCTCCTCTGTACC-3′	60	585
	R:5′-TCACCTCCTTCATGGTCTCC-3′		
*APOE* (RT-PCR)	F:5′-GCCACCCTGAGTACCCAG-3′	58	119
	F:5′-ATCTTGTCCAGGCGGTCC-3′		
*GAPDH* (RT-PCR)	F:5′-GGCCTCCAAGGAGTAAGGTC-3′	58	124
	F:5′-CGGGAGATTCTCAGTGTGGT-3′		

**Table 5:** Population genetic analysis of *APOE* gene in goats.

Genotype	Genotype Frequency	Allele	Allele Frequency	χ^2^	PIC
AA	12.97%	A	33.47%	1.58 (*p* = 0.21)	0.35
AG	41.00%	G	66.53%		
GG	46.03%				

**Table 6:** Association of *APOE* allele with body size traits in goats.

Body Size Traits	Allele	Present	Absent	*p*-Value
Body weight (kg)	A	29.24 ± 0.33	28.19 ± 0.35	0.027
	G	28.49 ± 0.27	29.99 ± 0.58	0.020
Heart girth (cm)	A	72.31 ± 0.52	71.05 ± 0.55	0.095
	G	71.40 ± 0.41	73.29 ± 0.91	0.059
Wither height (cm)	A	58.49 ± 0.63	57.10 ± 0.63	0.128
	G	57.78 ± 0.47	57.93 ± 1.21	0.907
Body length (cm)	A	54.43 ± 0.74	52.42 ± 0.67	0.039
	G	52.66 ± 0.57	56.28 ± 1.23	0.010
Circumference of cannon bone (cm)	A	9.81 ± 1.51	8.04 ± 1.56	0.401
	G	7.00 ± 1.79	9.37 ± 1.24	0.445

**Table 7:** Association of *APOE* genotype with body size traits in goat.

Body Size Traits	Genotype	Mean ± Standard Error	*p*-Value
Body weight (kg)	AA (*n* = 29)	30.02 ± 0.58 ^a^	0.024
	AG (*n* = 92)	28.91 ± 0.39 ^ab^	
	GG (*n* = 103)	28.17 ± 0.34 ^b^	
Heart girth (cm)	AA (*n* = 29)	73.32 ± 0.91	0.101
	AG (*n* = 92)	71.86 ± 0.62	
	GG (*n* = 103)	71.04 ± 0.55	
Wither height (cm)	AA (*n* = 29)	58.00 ± 1.20	0.282
	AG (*n* = 92)	58.66 ± 0.73	
	GG (*n* = 103)	57.10 ± 0.63	
Body length (cm)	AA (*n* = 29)	56.33 ± 1.22 ^a^	0.020
	AG (*n* = 92)	53.42 ± 0.89 ^ab^	
	GG (*n* = 103)	52.27 ± 0.66 ^b^	
Circumference of cannon bone (cm)	AA (*n* = 29)	9.87 ± 0.52	0.635
	AG (*n* = 92)	8.54 ± 0.91	
	GG (*n* = 103)	6.98 ± 0.80	

Values within the same column with different superscript letters differ significantly (*p* < 0.05).

**Table 8:** Association of *APOE* allele with body size traits in male and female goats.

Body Size Traits	Allele	Male			Female		
		Present	Absent	*p*-Value	Present	Absent	*p*-Value
Body weight (kg)	A	28.70 ± 0.64	27.86 ± 0.57	0.288	29.78 ± 0.37	28.54 ± 0.46	0.038
	G	28.07 ± 0.47	29.66 ± 1.35	0.258	28.98 ± 0.32	30.42 ± 0.63	0.046
Heart girth (cm)	A	71.78 ± 1.17	71.44 ± 1.05	0.817	73.17 ± 0.05	71.17 ± 0.62	0.013
	G	71.31 ± 0.86	74.11 ± 1.43	0.272	72.02 ± 0.45	73.64 ± 0.84	0.092
Wither height (cm)	A	58.80 ± 1.27	57.50 ± 0.99	0.404	58.18 ± 0.71	56.88 ± 0.88	0.244
	G	57.93 ± 0.85	58.44 ± 1.45	0.843	57.71 ± 0.61	57.53 ± 1.33	0.903
Body length (cm)	A	54.09 ± 0.89	52.54 ± 0.69	0.159	54.66 ± 1.53	51.86 ± 1.47	0.226
	G	52.80 ± 0.59	56.00 ± 1.68	0.075	52.39 ± 1.16	55.92 ± 2.21	0.188
Circumference of cannon bone (cm)	A	11.26 ± 1.93	8.85 ± 1.42	0.418	8.09 ± 0.25	7.47 ± 0.29	0.124
	G	7.11 ± 2.71	10.71 ± 1.65	0.467	7.73 ± 0.22	8.18 ± 0.44	0.384

**Table 9:** Association of *APOE* genotype with body size traits in male and female goats.

Body Size Traits	Genotype	Male		Female	
		Mean ± Standard Error	*p*-Value	Mean ± Standard Error	*p*-Value
Body weight (kg)	AA	29.70 ± 0.35	0.402	30.42 ± 0.63 ^a^	0.048
	AG	28.45 ± 0.71		29.44 ± 0.47 ^ab^	
	GG	27.83 ± 0.57		28.54 ± 0.46 ^b^	
Heart girth (cm)	AA	74.10 ± 1.44	0.544	73.64 ± 0.83 ^a^	0.037
	AG	71.19 ± 1.29		72.90 ± 0.64 ^ab^	
	GG	71.39 ± 1.05		71.17 ± 0.62 ^b^	
Wither height (cm)	AA	58.57 ± 1.46	0.704	57.51 ± 1.33	0.427
	AG	58.87 ± 1.44		58.44 ± 0.83	
	GG	57.51 ± 0.99		56.87 ± 0.89	
Body length (cm)	AA	56.09 ± 1.69	0.145	55.96 ± 2.25	0.363
	AG	53.47 ± 0.98		53.47 ± 2.16	
	GG	52.50 ± 0.69		51.85 ± 1.49	
Circumference of cannon bone (cm)	AA	11.31 ± 1.95	0.646	8.13 ± 0.43	0.315
	AG	9.42 ± 1.72		8.07 ± 0.35	
	GG	6.92 ± 2.74		7.47 ± 0.29	

Values within the same column with different superscript letters differ significantly (*p* < 0.05).

## Data Availability

The authors affirm that all of the data necessary for confirming the conclusions of this article are present within the article, figures and tables.
